# Assessment of Pathological Response of Breast Carcinoma in Modified Radical Mastectomy Specimens after Neoadjuvant Chemotherapy

**DOI:** 10.1155/2015/536145

**Published:** 2015-11-30

**Authors:** Dhanya Vasudevan, P. S. Jayalakshmy, Suresh Kumar, Siji Mathew

**Affiliations:** ^1^Department of Pathology, Pushpagiri Institute of Medical Sciences and Research Centre, Tiruvalla, Kerala 689101, India; ^2^Department of Pathology, Government Medical College, Thrissur, Kerala 680596, India; ^3^Department of Oncology, Government Medical College, Alappuzha, Kerala 688005, India; ^4^Department of Pathology, Government Medical College, Alappuzha, Kerala 688005, India

## Abstract

*Aim*. Paclitaxel based neoadjuvant chemotherapy regimen (NAT) in the setting of locally advanced breast cancer (LABC) can render inoperable tumor (T4, N2/N3) resectable. The aim of this study was to assess the status of carcinoma in the breast and lymph nodes after paclitaxel based NAT in order to find out the patient and the tumor characteristics that correspond to the pathological responses which could be used as a surrogate biomarker to assess the treatment response.* Materials and Methods*. Clinical and tumor characteristics of patients with breast carcinoma (*n* = 48) were assessed preoperatively. These patients were subjected to modified radical mastectomy after 3 courses of paclitaxel based NAT regimen. The pathological responses of the tumor in the breast and the lymph nodes were studied by using Chevallier's system which graded the responses into pathological complete response (pCR), pathological partial response (pPR), and pathological no response (pNR).* Results*. Our studies showed a pCR of 27.1% and a pPR of 70.9% . Clinically small sized tumors (2–5 cms) and Bloom Richardson's grade 1 tumors showed a pCR. Mean age at presentation was 50.58 yrs. 79.2% of cases were invasive ductal carcinoma NOS; only 2.1% were invasive lobular carcinoma, their response to NAT being the same. There was no downgrading of the tumor grades after NAT. Ductal carcinoma in situ and lymphovascular invasion were found to be resistant to chemotherapy. The histopathological changes noted in the lymph nodes were similar to that found in the tumor bed.* Discussion and Conclusion*. From our study we conclude that histopathological examination of the tumor bed is the gold standard for assessing the chemotherapeutic tumor response. As previous studies have shown pCR can be used as a surrogate biomarker to assess the tumor response.

## 1. Introduction

Breast carcinoma is the most common non-skin malignancy in women second to lung cancer as a cause of cancer deaths.

It has been estimated that out of all new cancer cases detected, 25% cases are breast carcinoma which also accounts for 15% of all cancer deaths among females [1]. According to GLOBOCAN 2012, the estimated cases of breast cancer worldwide are 1,676,600 with 521,900 deaths. In the developing countries the estimated new cases related to breast cancer were 882,900 and deaths were 324,300 [1]. The surveillance epidemiology and end results programme (SEER), India, Stat Fact Sheet states that estimated new cases of breast cancer in 2015 are 231840, 14% of all new cancer cases detected, and the estimated deaths were 40290, 6–8% of all cancer deaths [2].

The number of new cases in India, as per GLOBOCAN 2000, was 11,5251, with the mortality rates being 53592 [3]. These data clearly indicate that there has been an increase in incidence of breast cancer.

The addition of adjuvant chemotherapy to standard breast carcinoma treatment has shown improving its outcome substantially [4]. The arguments offered are that the neoadjuvant chemotherapy (NAT) downstages the tumor, enables to monitor the treatment efficacy and makes it possible to detect and treat the micrometastasis [5, 6]. It is possible to predict the prognosis in these patients on an individual basis by assessing the morphological parameters like a decrease in the tumor volume and inflammatory cell response.

In general, the histological responses to systemic chemotherapy can be correlated with the extent of clinical responses [7].

A study conducted by Montagna et al., 2010, states that pathological complete response (pCR) to neoadjuvant chemotherapy is the best predictor of overall survival (OS) [8].

The pathological response to paclitaxel was studied by Krishnan et al., where pCR was used as a surrogate marker for assessing the overall survival. They found that the overall pCR rate was 13.7% but with combination chemotherapy of anthracyclines and taxanes, higher rates (14.2%) could be attained [9].

We used Chevallier's system to assess the pathological responses to paclitaxel; our study showed a pathological complete response (pCR) in 27.1% of cases and a partial pathological response (pPR) in 70.9%.

The study conducted by Kulka et al. which aimed to study the breast cancer subtypes likely to respond to NAT also used the Chevallier system; 13 out of 92 cases showed a pCR (14.1%). Their study also states that pCR was associated with better OS (*p* = 0.050) [10].

The greatest histopathological alterations are usually found in patients who appear to have a complete resolution of their neoplasm clinically [11, 12].

A study conducted by Vinnicombe et al. reported that out of 8 patients who were reported to have a complete resolution as per mammography 5 had residual neoplasm [13].

Prognosis of the patient in terms of 5-year survival rates is related to the completeness of the response and it is favourable for those patients in whom there was the least evidence of the residual neoplasm after NAT.

Thus, the histopathological examination of the tumor bed would be the gold standard to decide the presence of neoplasm after NAT.

## 2. Materials and Methods

Eligibility criteria in this study were patients (*n* = 48) with locally advanced breast cancer with axillary lymph node involvement and absence of distant metastasis.

Cases of previous lumpectomy were excluded from the study. Consent for the study was obtained from the Ethical Committee.

In this study, all patients were diagnosed to have breast carcinoma by either cytology or trucut biopsy. Prior to being subjected to chemotherapy the clinical data and the pretherapeutic clinical size of the tumor were recorded in all cases. Prechemotherapeutic tumor grading was done either from cytology smears or trucut biopsy sections (Figure 2).

The histological grading was done by Bloom Richardson grading system. The cytological grading was done by Robinson's method.

Patients received 3 courses of paclitaxel based chemotherapy (adriamycin, cyclophosphamide, and paclitaxel) and underwent modified radical mastectomy with axillary lymph node dissection (MRM).

The postchemotherapeutic changes were studied from the MRM specimens. The tumor was studied from paraffin embedded H&E sections obtained from the tumor bed. In complete responders, the tumor bed was identified from the areas of fibrosis by multiple sampling. In partial responders and nonresponders, the tumor was evident grossly or microscopically and adequate sampling of the area was done. Tumor grade, histological type, and response to chemotherapy (by Chevallier's method) were assessed.

*Bloom Richardson Grading*. See Table 1:

 Grade 1: score of 3–5 (well differentiated).
 Grade 2: score of 6-7 (moderately differentiated).
 Grade 3: score of 8-9 (poorly differentiated).

**Figure 1:**
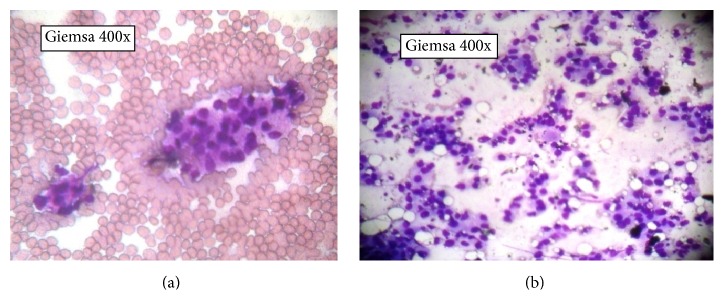
(a and b) Grade 1 tumor and Grade 2 tumor as per Robinson's grading.

**Figure 2:**
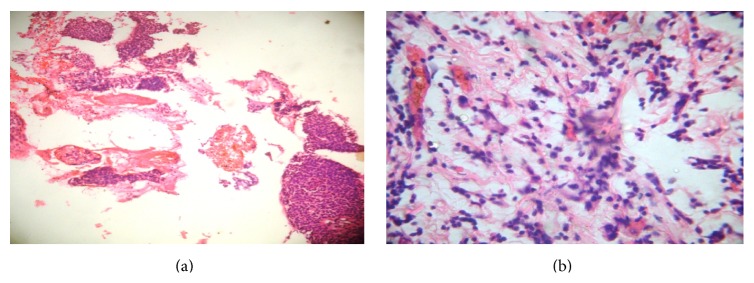
(a) Photomicrographs of trucut biopsy before NAT (H&E ×100). (b) Same case after receiving NAT showing a pCR (H&E ×400).

*Cytological Grading from the FNAC Smears Was Done by Robinson's Grading System* (Figures 1(a) and 1(b)). See Table 2:

 Grade 1: score of 6–11.
 Grade 2: score of 12–14.
 Grade 3: score of 15–18.

### 2.1. Assessment of the Pathological Tumor Response

The histopathological evidence of the chemotherapeutic response was graded from the H&E sections on the basis of the parameters used by Chevallier in his study [14].

According to the Chevallier system we have the following:

 pCR (pathological complete response): it was defined as the disappearance of all the tumor or DCIS in breast with no invasive carcinoma and negative lymph nodes.
 pPR (pathological partial response): it was defined as presence of invasive carcinoma with stromal alterations.
 pNR (pathological no response): it was defined as little modification in the original tumor appearance.

Only the invasive carcinomas and the lymph nodes were graded on the above-mentioned criteria.

The presence of lymphovascular emboli and presence of ductal carcinoma in situ were noted separately.

## 3. Analysis of the Results

All the data were analysed with the software of SPSS v14 for windows.

Baseline sociodemographic characteristics and microscopic findings were compiled as numbers and percentages.

Response to neoadjuvant chemotherapy was also found out and expressed as numbers and percentages.

Possible risk factors affecting tumor response and lymph node response were tested using tests of proportions (Pearson's chi square test and Fischer's exact test whichever was applicable) and *p* value was calculated. Odd's ratio and 95% confidence interval CI were also calculated from the data.

## 4. Results

In our study, majority of the patients were of the age group between 40 and 59 yrs, the mean age of presentation being 50.58 yrs (with a standard deviation of 10.94). All the 48 cases studied were females. Eight cases (16.7%) had a positive family history for breast cancer. Majority of the cases had a clinical size of 2 cm–5 cm at the time of presentation (66.7%), mean clinical size being 3.75 cm (with a standard deviation of 2.36) (Table 3).

Microscopic findings showed that most of the cases of pPR were of the invasive ductal carcinoma NOS (79.2%), the rest being invasive lobular carcinoma (2.1%) (Table 4).

In the postoperative grading of the tumor, based on Bloom Richardson's grading system 13 cases of pPR were of grade 1 (27.1%), 21 cases were grade 2 tumors (43.8%), and only 1 case was grade 3 tumor (2.1%). 13 cases showed a pCR (27.1%) (Table 4).

Ductal carcinoma in situ (DCIS) was noted in 13 cases (27.1%) (Table 4). Lymphovascular invasion was noted in 13 cases (27.1%) (Table 4).

When the tumor response to NAT was analysed we found that 13 cases (27.1%) showed a pathological complete response whereas 35 cases (72.9%) showed a pathological partial response (Table 5).

When the possible risk factors for a pathological partial response were analysed it was found that 27 cases were of the age group up to 59 yrs (73%) and 9 cases were 60 yrs of age and above (81.8%). 62.5% had a positive family history. In 26 cases the preoperative clinical size was up to 5 cm (70.3%) and in 10 cases (90.9%) the clinical size was more than 5 cm. The preoperative cytological grading showed that 22 cases (81.5%) were of grade 2 tumors whereas 14 cases (66.7%) were of grade 1 tumors (Table 6).

11 cases (84.6%) had ductal carcinoma in situ with a *p* value of 0.348 and OR of 2.20 (0.41–11.75).

92.1% were invasive ductal carcinoma NOS; this was statistically found to be significant with a *p* value of <0.001 (Table 6).

13 cases (100%) of pPR showed presence of lymphovascular invasion, which was statistically found to be significant with a *p* value of 0.015.

As listed in Table 6, the risk factors for pathological complete response showed the following results.

27% of cases belonged to the age group below 59 yrs and only 2 cases (18.2%) were of the age group above 60 yrs.

37.5% of cases had a positive family history for breast cancer. 11 cases (29.7%) had a tumor size of less than 5 cm preoperatively and only 9.1% had a tumor size of above 5 cm, which showed a *p* value of 0.165 and 95% CI of 0.23 (0.02−2.07).

33.3% of cases were grade 1 tumors and 18.5% were grade 2 tumors with 95% CI of 0.45 (0.12–1.71).

DCIS was present only in 2 cases with pCR (15.4%); *p* value when calculated was 0.348 with a confidence interval of 2.20 (0.41–11.75).

Lymphovascular invasion was not seen in any case which showed pCR; this was found to be statistically significant with a *p* value of 0.015.

On analysing the risk factors affecting the lymph node response, it was noted that when the tumor size was less than 5 cm, 15 cases (40.5%) showed a pCR and 59.5% cases showed a pPR (95% CI of 0.83 (0.20−3.37)) (Table 7).

10 cases (47.6%) that showed a pCR in the lymph nodes were grade 1 tumors and 9 cases (33.3%) were grade 2 tumors. Of the cases that showed a pPR in the lymph nodes, 11 cases (52.4%) were grade 1 tumors and 18 cases (66.7%) were grade 2 tumors. *p* value for which was 0.315 and 95% CI 0.55 (0.17–1.77) (Table 7).

Lymphovascular invasion was not seen in any case that showed a pCR and in 13 cases (100%) of pPR. This data was found to be statistically significant with a *p* value of 0.001 (Table 7).

## 5. Discussions and Conclusions

### 5.1. Characteristics of Patients and Tumor

In our study the mean age of patients at diagnosis was 50.58 yrs with a standard deviation of 10.94. In a study conducted by Saxena et al. the mean age of presentation of breast cancer in the Indian population was 47.8 yrs [15] and as per the study conducted by Chin et al. the mean age at presentation was 52 yrs [16].

In our study the mean clinical tumor size at the diagnosis was 3.75 cm, with a standard deviation of 2.36; it was also noted that the younger patients responded with a pCR (Table 3).

In the study of Chin et al. majority of the tumors presented in T2 stage (TNM staging), which is in accordance with our study. Their study also recommends that these patients could be candidates for NAT based on international standards [17]. A multivariate analysis study conducted by Galal et al. showed that initial tumor size < 5 cm, absence of ductal carcinoma in situ, and absence of vascular invasion were the best predictors of tumor response to chemotherapy [18]. Their study states that clinically small sized tumors responded better to chemotherapeutic regime [18]. Study conducted by Adam et al. showed that extremes of age had tendency to develop a higher grade tumor. In our study there was only a single case of grade 3 tumor and the age group corresponded to 40 yrs; majority of the cases were low grade tumors even in the elderly (60–80 yrs).

### 5.2. Response of Tumor

Out of the total 48 cases studied there was a complete pathological response (pCR) in 27.1% of cases. 70.9% showed a partial pathological response (pPR). 58% cases that showed a pCR were grade 1 tumors. In tumors that showed a pPR, 27.1% were grade 1 tumors, 27.1% were grade 2 tumors, and 2.1% were grade 3 tumors (Table 5).

Majority were grade 2 tumors out of which 81% showed pPR and 18.5% showed pCR. Out of the 21 cases of grade 1 tumors 66.7% showed pPR and 33% showed pCR.

A pCR of 19–31% has been found in large prospective studies conducted by Smith et al. [19] and Baer et al. [20]; however the study conducted by Chin et al. found a lower rate of pCR of 10% [16]. Studies of Fayanju et al. showed a pCR of 40% [21].

Majority of the cases (99%) were of invasive ductal carcinoma NOS; only 1 case of lobular carcinoma was encountered during the study. In a published series by Elston and Ellis the most common type of breast carcinoma was those of invasive duct carcinoma NOS which comprised about 40–75% [22]. According to Galal et al. invasive duct carcinoma NOS was found in 88% of their cases and invasive lobular carcinoma was about 12% [18].

There were no differences in the tumor grades assessed prechemotherapeutically and postchemotherapeutically (Wilcoxon signed rank test of significance <0.001). Study by Frierson and Fechner noted that there were no differences in the histological grading of pretreatment and postchemotherapy surgical specimens [23].

In our study DCIS and lymphovascular invasion were found to be resistant to chemotherapy. Our study showed a strong association between lymphovascular invasion and tumor response suggesting that the presence of lymphovascular invasion could indicate a poor tumor response (*p* value of 0.015). DCIS was consistently present in 84% of the cases with pCR indicating that it was resistant to chemotherapy. Study by Galal et al. showed that 86% of tumors with DCIS had a poor response to treatment [18].

### 5.3. Tumor Response in Lymph Nodes

Patients with a complete response in both breast and lymph nodes have significantly improved overall and disease-free survival [24]. In our study, in patients with pCR the histopathological findings were similar in the breast and lymph nodes which showed presence of histiocytes, giant cells, inflammatory infiltrate, and so forth.

**Figure 3:**
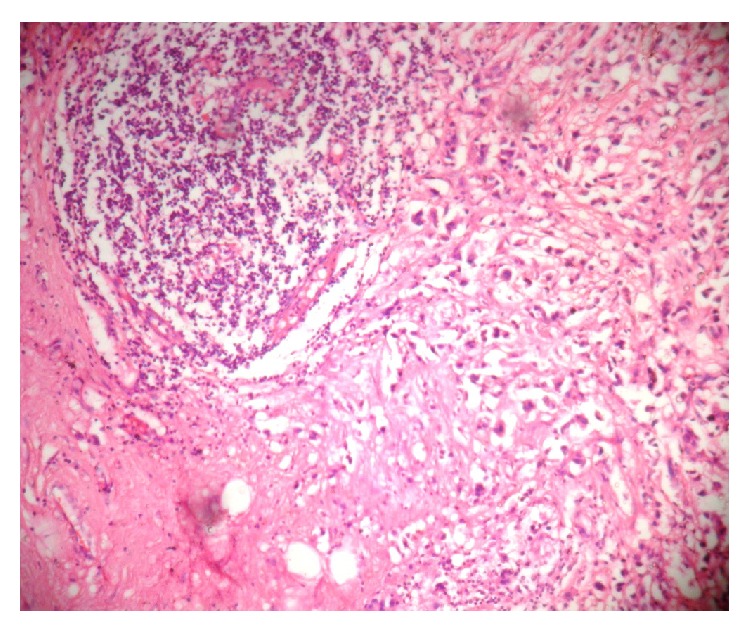
Cases of pPR showing inflammatory reaction (H&E ×400).

**Figure 4:**
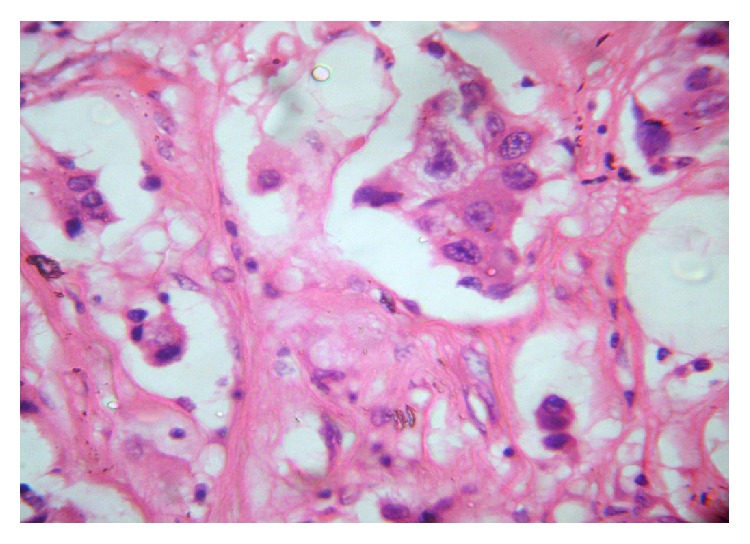
Photomicrograph of a case with pPR (H&E ×400).

In cases of pPR, individual nodes in a single case showed variable responses. In these cases, nodes which showed a complete pathological response did not show any evidence of tumor but instead showed areas of fibrosis, calcification, and lymphoplasmacytic inflammatory response, whereas some nodes in the same case did not show any response to the treatment given. pCR was noted only in 7% of cases whereas pPR was noted in 93% cases. As per National Surgical Adjuvant Breast and Bowel project (NSABP 18) during a period of 9 yr follow-up, patients with negative nodes or micrometastasis who were not treated with chemotherapy before surgery had identical survival, whereas those patients with macrometastasis had a significantly worse survival [25] (Figures 3 and 4).

## 6. Conclusion

From our study we conclude that histopathological examination of the tumor bed is the gold standard for assessing the chemotherapeutic tumor response. Young patients with a clinically T2 tumor size and lower tumor grades were good responders to chemotherapy. Tumor grades were not downgraded after chemotherapy. pCR can be used as a surrogate biomarker to assess the tumor response to paclitaxel based chemotherapy regimen, as pCR has been stated as an indicator of overall survival rate by various studies. Ductal carcinoma in situ and lymphovascular emboli were resistant to chemotherapy and these patients may have a poor prognosis.

## Figures and Tables

**Table 1:** Bloom Richardson grading.

	Score 1	Score 2	Score 3
Tubule formation	>75%	10–75%	<10%
Nuclear pleomorphism	Mild	Moderate	Marked
Mitotic figures	<7/10 HPF	8–14/10 HPF	>15/10 HPF

**Table 2:** Robinson's grading system.

	Score 1	Score 2	Score 3
Cellular cohesion	Clusters	Singly	
Cell size	1-2 times the RBC	3-4 times the RBC	>5 times the RBC
Cell uniformity	Monomorphic	Mild pleomorphism	Markedly pleomorphic
Nuclear margins	Smooth	Folds	Buds or tufts
Nuclear chromatin	Vesicular	Granular	Clumps or cleaved

**Table 3:** Baseline sociodemographic characteristics.

Characteristic	Number	Percentage
Age		
Up to 39	5	10.4
40 to 59	32	66.7
60 and above	11	22.9
Sex		
Female	48	100
Male	0	0
Family history		
Yes	8	16.7
No	40	83.3
Specimen size		
Up to 2 cm	5	10.4
2 cm to 5 cm	32	66.7
More than 5 cm	11	22.9
Pre-op FNA grade (Robinsons)		
Grade 1	21	43.8
Grade 2	27	56.2

**Table 4:** Microscopic findings on examination of the resected specimen.

Characteristic	Number	Percentage
Type of carcinoma		
pCR	9	18.8
Invasive ductal carcinoma NOS	38	79.2
Invasive lobular carcinoma	1	2.1
Post-op grade (Bloom Richardson)		
Grade 0	13	27.1
Grade 1	13	27.1
Grade 2	21	43.8
Grade 3	1	2.1
Ductal carcinoma in situ (DCIS)		
Yes	13	27.1
No	35	72.9
Lymph node invasion		
Yes	13	27.1
No	35	72.9

**Table 5:** Percentage of tumors with the tumor responses and frequencies.

Grades	Frequency	Percentage
pCR	13	27.1%
pPR		
Gr1	13	27.1%
pPR		
Gr2	21	43.8%
pPR		
Gr3	1	2.1%

Total	48	100%

**Table 6:** Factors affecting the tumor response.

Characteristic	Tumor partial response (pPR)	Tumor complete response (pCR)	*p*	OR (95% CI)
Age				
Up to 59	27 (73%)	10 (27%)	0.552	0.60 (0.11 to 3.26)
60 and above	9 (81.8%)	2 (18.2%)		
Family history				
Yes	5 (62.5%)	3 (37.5%)	0.371	0.48 (0.09 to 2.42)
No	31 (77.5%)	9 (22.5%)		
Specimen size				
Up to 5 cm	26 (70.3%)	11 (29.7%)	0.165	0.23 (0.02 to 2.07)
More than 5 cm	10 (90.9%)	1 (9.1%)		
FNA grade				
Grade 1	14 (66.7%)	7 (33.3%)	0.240	0.45 (0.12 to 1.71)
Grade 2	22 (81.5%)	5 (18.5%)		
DCIS				
Yes	11 (84.6%)	2 (15.4%)	0.348	2.20 (0.41 to 11.75)
No	25 (71.4%)	10 (28.6%)		
Type of cancer				
pCR	1 (11.1%)	8 (88.9%)	<0.001^#^	—
Invasive ductal carcinoma NOS	35 (92.1%)	3 (7.9%)		
Invasive lobular carcinoma	0	1 (100%)		
Lymphovascular invasion				
Yes	13 (100%)	0	0.015^#^	—
No	23 (65.7%)	12 (34.3%)		

^#^Statistically significant.

**Table 7:** Factors affecting lymph node response.

Characteristic	Lymph node partial response	Lymph node complete response	*p*	OR (95% CI)
Age				
Up to 59	22 (59.5%)	15 (40.5%)	0.804	0.83 (0.20 to 3.37)
60 and above	7 (63.6%)	4 (36.4%)		
Family history				
Yes	6 (75%)	2 (25%)	0.356	2.21 (0.39 to 12.36)
No	23 (57.5%)	17 (42.5%)		
Specimen size				
Up to 5 cm	22 (59.5%)	15 (40.5%)	0.804	0.83 (0.20 to 3.37)
More than 5 cm	7 (63.6%)	4 (36.4%)		
FNA grade				
Grade 1	11 (52.4%)	10 (47.6%)	0.315	0.55 (0.17 to 1.77)
Grade 2	18 (66.7%)	9 (33.3%)		
DCIS				
Yes	8 (61.5%)	5 (38.5%)	0.923	1.06 (0.28 to 3.93)
No	21 (60%)	14 (40%)		
Type of cancer				
pCR	2 (22.2%)	7 (77.8%)	0.012^#^	—
Invasive ductal carcinoma NOS	27 (71.1%)	11 (28.9%)		
Invasive lobular carcinoma	0	1 (100%)		
Lymphovascular invasion				
Yes	13 (100%)	0	0.001^#^	—
No	16 (45.7%)	19 (54.3%)		

^#^Statistically significant.
